# Integrated physiological and metabolomic responses reveal mechanisms of Cd tolerance and detoxification in kenaf (*Hibiscus cannabinus* L.) under Cd stress

**DOI:** 10.3389/fpls.2024.1332426

**Published:** 2024-08-08

**Authors:** Wajid Saeed, Samavia Mubeen, Jiao Pan, Muzammal Rehman, Wangqiang Fang, Dengjie Luo, Pingwu Liu, Yun Li, Peng Chen

**Affiliations:** ^1^ Guangxi Key Laboratory of Agro-environment and Agric-products Safety, Key Laboratory of Plant Genetics and Breeding, College of Agriculture, Guangxi University, Nanning, China; ^2^ Sanya Nanfan Research Institute of Hainan University, Sanya, China

**Keywords:** *Hibiscus cannabinus* L. (kenaf), Cd stress, metabolites, stress physiology, heavy metal tolerance

## Abstract

**Introduction:**

Cadmium (Cd) is a highly toxic trace element that occurs in large quantities in agricultural soils. The cultivation of industrial crops with high phytoremediation potential, such as kenaf, could effectively reduce soil Cd contamination, but the mechanisms of toxicity, tolerance, and detoxification remain unclear.

**Methods:**

In this study, the effects of different Cd concentrations (0, 100, 250, and 400 µM) on growth, biomass, Cd uptake, physiological parameters, metabolites and gene expression response of kenaf were investigated in a hydroponic experiment.

**Results and discussion:**

The results showed that Cd stress significantly altered the ability of kenaf to accumulate and transport Cd; increased the activity of hydrogen peroxide (H_2_O_2_), superoxide anion (O_2_^−^), and malondialdehyde (MDA); reduced the activities of superoxide dismutase *(*SOD) and catalase (CAT); and decreased the content of photosynthetic pigments, resulting in significant changes in growth and biomass production. Exposure to Cd was found to have a detrimental effect on the ascorbate–glutathione (AsA–GSH) cycle in the roots, whereas it resulted in an elevation in AsA levels and a reduction in GSH levels in the leaves. The increased content of cell wall polysaccharides under Cd stress could contribute to Cd retention in roots and limited Cd transport to above-ground plant tissues. Metabolomic analyses revealed that alanine, aspartate, and glutamate metabolism, oxidative phosphorylation, ABC transporter, and carbon metabolism were the major metabolic pathways associated with Cd stress tolerance. Cd stress increased gene expression of *IRT1* and *MTP1* in roots, which resulted in kenaf roots accumulating high Cd concentrations. This study extends our knowledge of the factors regulating the response of kenaf to Cd stress. This work provided a physiological and metabolomic perspective on the mechanism controlling the response of kenaf to Cd stress.

## 1 Introduction

Cadmium (Cd) is a highly hazardous, non-essential metal ion of significant concern in agricultural soils due to its high mobility, water solubility, and long biological half-life (>20 years) (Bisht and Garg, 2022). Major contributors to Cd contamination of agricultural soils include industrial operations, mining activities, smelting processes, the application of fertilizers, and the utilization of sewage sludge (Huang et al., 2017). High Cd concentrations in crops can lead to increased health risks for animals and humans as exemplified by kidney and bone disease (*itai-itai*) in humans famously recorded in Japan (Huang et al., 2017). The toxicity of Cd in plants can lead to several detrimental effects, such as diminished growth and yield, poor photosynthesis and respiration, hindered nutrient uptake and metabolism, and decreased enzyme functionality. Excessive metal accumulation within the nucleus can lead to DNA damage in cases of greater severity (Haider et al., 2021; Mubeen et al., 2023). Cadmium toxicity differs according to plant species, growth stage, and Cd concentration in the soil (Bisht and Garg, 2022). At the cellular level, the toxicity of Cd is manifested through the initiation of oxidative stress, which subsequently disrupts the normal functioning of cellular membranes (Dutta et al., 2018). During oxidative stresses, the accumulation of reactive oxygen species (ROS) such as hydrogen peroxide (H_2_O_2_), malondialdehyde (MDA), and superoxide anion (O_2_^−^) has been observed to increase (Hasanuzzaman et al., 2020; Cao et al., 2024); to counteract the elevated levels of ROS, plants activate their defense system, which encompasses enzymatic components such as superoxide dismutase (SOD), peroxidase (POD), and catalase (CAT), as well as non-enzymatic components including ascorbate–glutathione (AsA–GSH) and osmolytes (proline, glycine betaine, and sugars) (Ghori et al., 2019; Tanveer and Ahmed, 2020).

There are several ways to remediate heavy metal pollution from the soil. In comparison to physical and chemical methods, phytoremediation by plants emerges as a highly promising approach due to its high efficiency, low cost, absence of secondary contaminants, and on-site applicability (Yan et al., 2020). Nevertheless, this technique is still in the research and development phase, with various technical challenges that need to be addressed, including impeded growth activities such as decreased biomass and increased susceptibility to Cd in plants utilized in phytoremediation (Haller and Jonsson, 2020). Thus, cultivating species with high biomass production and adaptability to contaminated soils, while ensuring the absence of health hazards, may be a cost-effective and environmentally sustainable approach to remediate Cd polluted soil.

Kenaf (*Hibiscus cannabinus* L.) belongs to the Malvaceae family, is a versatile fiber crop, and has been identified as a potential phytoremediation candidate due to its capacity to tolerate heavy metals in soil (Zhao et al., 2022). This is attributed to its high biomass production, deep-root system, and ability to accumulate heavy metals such as Cd, copper (Cu), and chromium (Cr) (Saleem et al., 2020; Chen et al., 2021; Tang et al., 2022). Cao et al. (2024) observed that the kenaf shoots could accumulate a maximum concentration of 546.94 mg/kg of Cd when cultivated in a hydroponic solution for 15 days, whereas kenaf absorbed 2.49 mg/kg of Cd without any apparent detrimental effects when grown in sludge for 3 months (Arbaoui et al., 2013). Although studies on Cd exposure have greatly improved our knowledge of the mechanisms underlying plant responses to heavy metals, there is currently very little information specifically addressing the effects of Cd stress on kenaf. To optimize the use of kenaf as a phytoremediation candidate for Cd contaminated soils, it is imperative to gain a comprehensive understanding of the underlying mechanisms that enable this plant to tolerate Cd stress, particularly about kenaf genotypes that exhibit Cd accumulation.

In this study, we investigated the mechanisms of Cd stress tolerance in kenaf plants using physiological, metabolomic, and gene expression methods. Considering that the majority of metal-polluted soils are usually contaminated with multiple metals, which may alter the outcome of the remediation process of Cd by kenaf, we planned this experiment in a hydroponic environment to avoid competition with other metals. The objectives of this study were as follows: a) to investigate the uptake and transport of Cd in kenaf under different levels of Cd-induced stress, b) to evaluate the physiological and biochemical alterations in leaves and roots of kenaf under different levels of Cd stress, c) to identify specific metabolites that contribute to Cd stress tolerance and their key pathways, and d) to elucidate the regulatory mechanism involved in Cd transport and tolerance. This study will provide a theoretical basis for future research by clarifying the mechanisms of kenaf tolerance and detoxification in response to varying levels of Cd stress.

## 2 Material and methods

### 2.1 Plant material and growth conditions

Seeds of kenaf cultivar CP085 (designated with the code LT) were surface sterilized with 1% sodium hypochlorite solution (NaClO) for 10 min and then extensively rinsed with deionized water. Seeds were placed in a growth chamber (model BA170421, Shanghai Fuma Laboratory Instrument Co., Ltd., Shanghai, China) to facilitate seed germination. Seedlings were transplanted into hydroponic trays containing a half-strength Hoagland nutrient solution (pH 6.0) 1 week after germination. To ensure good growth, the nutrient solution was renewed twice a week. After 2 weeks of hydroponic growth, the seedlings were exposed to a nutrient solution supplemented with different concentrations of CdCl_2_·2.5H_2_O (0, 100, 250, and 400 μM). Each treatment was repeated three times, with 18 seedlings per replicate. The experiment was conducted in an ecologically uncontrolled glasshouse with a day/night cycle of 12/12 hours, the temperature ranged from 15°C to 22°C, and the relative humidity remained between 65% to 75%. After 10 days of Cd treatment, leaves and roots were collected in triplicates and promptly frozen in liquid nitrogen. These samples were subsequently stored at a temperature of −80°C to facilitate further experiments.

### 2.2 Growth analysis and determination of chlorophyll pigments

After harvest, roots were rinsed with ultra-pure water and treated in 20 mM disodium ethylenediaminetetraacetic acid (Na_2_-EDTA) for 10 min to remove nutrients and Cd ions from the root surface. Roots and shoots were separated, and shoot length, diameter, leaf surface area (LSA), and fresh weight (FW) of shoots and roots were measured. After the samples were dried to a consistent weight in an oven at 65°C, the dry weight (DW) was measured. A chlorophyll meter (model SPAD-502, Konica Minolta Camera Co., Ltd., Tokyo, Japan) was used to determine chlorophyll content in leaves. The spectrophotometric protocol outlined by Anjum et al. (2017) was used to determine the amounts of photosynthetic pigments (chlorophyll *a*, chlorophyll *b*, total chlorophyll, and carotenoids) of kenaf leaves.

### 2.3 Quantification of Cd element

The powdered, dried tissues of roots and shoots were placed in HNO_3_/HClO_4_ (4:1, v/v) solution and heated to 200°C until complete digestion was achieved. Inductively coupled plasma–mass spectrometry (ICP-MS; NexIONTM 350X, PerkinElmer, Waltham, MA, USA) was used to measure Cd concentration after dilution with deionized water. A certified plant reference material (CRM) (GBW-07603, provided by the National Research Center for CRM, Beijing, China) with a known Cd concentration of 0.38 mg/kg was used to monitor the analysis accuracy. The following formula was used to calculate the bioconcentration factor (BCF) and translocation factor (TF) of Cd.

BCF = (*C_plant_*/*C_nutrient solution_*)

TF = (*C_shoot_*/*C_root_*).

### 2.4 Oxidative stress indicators and proline content measurement

#### 2.4.1 Superoxide anion

The O_2_^−^ content from leaves and roots (0.2 g) of kenaf was extracted using a 0.05 M phosphate buffer containing 2% PVP K-30 and 0.50% Triton X-100. The extraction solution was combined with 0.05 M phosphate buffer and 10 mM hydroxylamine hydrochloride solution before being heated at 25°C for 30 min. Following incubation, the reaction mixture was mixed with 58 mM 3-aminobenzenesulfonic acid and 7 mM 1-naphthylamine and heated at 25°C for a further 20 min. The absorbance of the reaction solution was measured at 530 nm (Wu et al., 2010).

#### 2.4.2 Hydrogen peroxide

The plant tissue samples (0.2 g) were homogenized in 50 mM phosphate buffer and then centrifuged at 6,000 × *g* for 25 min at 4°C. After 3 mL of supernatant was added to 0.1% titanium chloride (w/v) in 20% H_2_SO_4_, the absorbance of the reaction mixture was measured at 410 nm (Yao et al., 2015).

#### 2.4.3 MDA content

A 0.2 g sample of kenaf leaves and roots was homogenized in 5 mL of 0.1% trichloroacetic acid solution and centrifuged for 10 min at 10,000 × *g*. After 2 mL of 0.6% thiobarbituric acid was added to the supernatant, the samples were heated in a water bath at 100°C for 15 min, cooled in an ice bath for 10 min, and then centrifuged for 10 min at 10,000 × *g*. The absorbance of the supernatant was measured at wavelengths of 532 nm and 600 nm as described by Dad et al. (2020).

#### 2.4.4 Proline content

For proline content, 0.2 g kenaf tissue samples were homogenized in 3% sulfosalicylic acid solution, heated for 10 min in a water bath, and then centrifuged at 3,000 rpm for 10 min at 4°C. The supernatant was mixed with 60% (v/v) glacial acetic acid and 2.5% (w/v) ninhydrin, heated for 1 hour at 100°C, and immediately cooled in an ice bath to stop the reaction. After 4 mL of toluene was added to the reaction mixture, the absorbance of the reaction mixture was measured at 520 nm (Bates and Teare, 1973).

### 2.5 Investigation of enzymatic antioxidants activities

To further investigate the changes in enzymatic antioxidants activities, 0.2 g of fresh leaves and roots of kenaf was homogenized in 50 mmol sodium phosphate buffer supplemented with EDTA (0.5 mmol) and NaCl (0.15 mol). Following centrifugation of the homogenates at 12,000 × *g* for 10 min at 4°C, the supernatant was used to determine SOD, POD, and CAT activities according to the protocols reported by Wu et al. (2017).

### 2.6 Investigation of non-enzymatic antioxidants

The chilled samples were ground with liquid nitrogen before being homogenized in 5% tricarboxylic acid (TCA). The homogenate was centrifuged at 12,000 × *g* for 10 min at 4°C to estimate GSH and AsA levels. Total and reduced GSH levels were calculated following the method described by Salbitani et al. (2017). The content of oxidized glutathione (GSSG) was determined by subtracting the total GSH level from the reduced GSH level. The quantification of total ascorbic acid and reduced ascorbic acid (AsA) was conducted following the methodology outlined by Gillespie and Ainsworth (2007). The difference between the total AsA and the reduced AsA concentration was subtracted to determine the oxidized AsA (DHA).

### 2.7 Extraction of cell wall and its components determination

The crude cell wall from the roots of kenaf was extracted by the sequential use of ice-cold water, 80% ethanol, a mixture of methanol and chloroform in a 1:1 volume ratio, and acetone as described by Hu and Brown (1994). The three types of cell wall pectin, namely, water-soluble pectin (WSP), chelate-soluble pectin (CSP), and alkali-soluble pectin (ASP) were extracted from crude cell wall according to the method published by Redgwell and Selvendran (1986). The amount of uronic acid present in pectin was determined following the protocol of Blumenkrantz and Asboe-Hansen (1973). In addition, cellulose, hemicellulose 1 (HCI), and hemicellulose II (HCII) were extracted from crude cell wall samples as described by Gao et al. (2021). The phenol–sulfuric acid method was used to calculate the total sugar concentration of cellulose, HCI, and HCII (Dubois et al., 1956).

### 2.8 Quantitative real-time PCR analysis

In this study, quantitative real-time PCR (qRT-PCR) analyses of known Cd-inducible genes were performed to determine whether there are differential expression patterns of divalent heavy metal ion transporter genes, Cd-resistance genes, and antioxidant enzyme genes in response to varying Cd concentrations. Total RNA was isolated from kenaf leaves and roots using the EASYspin RNA Rapid plant kit (RA106). The reverse transcription of total RNA was carried out using TransScript One-Step gDNA Removal and cDNA Synthesis SuperMix (TransGen Biotech, Beijing, China). The qRT-PCR was run on a Bio-Rad CFX96 (Bio-Rad Laboratories, Hercules, CA, USA) with PerfectStart Green qPCR SuperMix (TransGen Biotech, Beijing, China). The experiment was performed using a final volume of 20 μL, and the amplification conditions were as follows: the DNA was first denatured at 94°C for 3 min and then 45 cycles of denaturation at 94°C for 10 seconds, followed by annealing and extension at 60°C for 30 seconds. The 2^−ΔΔCT^ approach was used to determine relative gene expression (Wei et al., 2019). The coding sequences (CDSs) of corresponding genes to design primer were derived from our earlier transcriptome data of kenaf (Cao et al., 2024), and primers for the corresponding genes and the reference gene *Histone3* were designed using Primer Premier 5.0 (**Supplementary Table 1**).

### 2.9 Metabolomic analysis

Kenaf leaves (1 g) from plants treated with 0, 100, and 400 μM Cd (three replicates per treatment) were homogenized in 1 mL water:acetonitrile (ACN):isopropanol (1:1:1, v/v) solution. The mixture was sonicated at 4°C for 30 min to extract the metabolites, and then the homogenate was centrifuged at 12,000 rpm for 20 min. The supernatant was kept at −20°C for precipitation and centrifuged again at 12,000 rpm for 20 min. The supernatant was then dried in a vacuum desiccator and reconstituted in 30% ACN for metabolite analysis. Data were collected by ultrahigh performance liquid chromatography–electrospray ionization–tandem mass spectrometry (UHPLC–ESI–MS/MS) (UPLC, Vanquish; MS, HFX). The sample at a volume of 2 μL was injected at a flow rate of 0.3 mL/min onto a UPLC@HSS T3 (Waters, Wilmslow, UK) column (100 * 2.1 mm diameter, thickness 1.8 μm) with a column temperature of 40°C. The mobile phase consisted of acetonitrile + 0.1% formic acid (solvent A) and water + 0.1% formic acid (solvent B). The elution gradient program was as follows: 0–1 min, 0% phase B; 1–9 min, 0%–95% phase B; 9–13 min, 95% phase B; 13–13.1 min, 95%–0%; 13.1–17 min, 0% phase B. Quality control (QC) samples were pooled and analyzed with the other samples to test instrument stability and repeatability. Raw data from the Q Exactive’s MS were acquired using Xcalibur 4.1 (Thermo Fisher Scientific, Waltham, MA, USA) and subsequently processed using Progenesis QI (Waters Corporation, Milford, MA, USA). Principal component analysis (PCA) and partial least-squares discriminant analysis (PLS-DA) were performed on the data using the R package. The significance of each metabolite was calculated using Student’s *t*-test at the univariate level for those having variable importance in the projection (VIP values) >1. *p*-Values less than 0.05 were considered statistically significant. Finally, the Kyoto Encyclopedia of Genes and Genomes (KEGG) database was used to perform pathway enrichment analysis of differential metabolites.

### 2.10 Data analysis

Univariate analyses with the Statistix 8.1 package were performed using one-way analysis of variance (ANOVA) for which the data are presented as arithmetic means with a standard error unless otherwise specified. The mean comparison between groups was performed following the least significant difference (LSD) test. The significance threshold was set at *p* < 0.05. R Studio, OriginPro, and Excel 2010 were used for visual presentation of the results.

## 3 Results

### 3.1 Effect of Cd stress on kenaf growth characteristics

**Figure 1:**
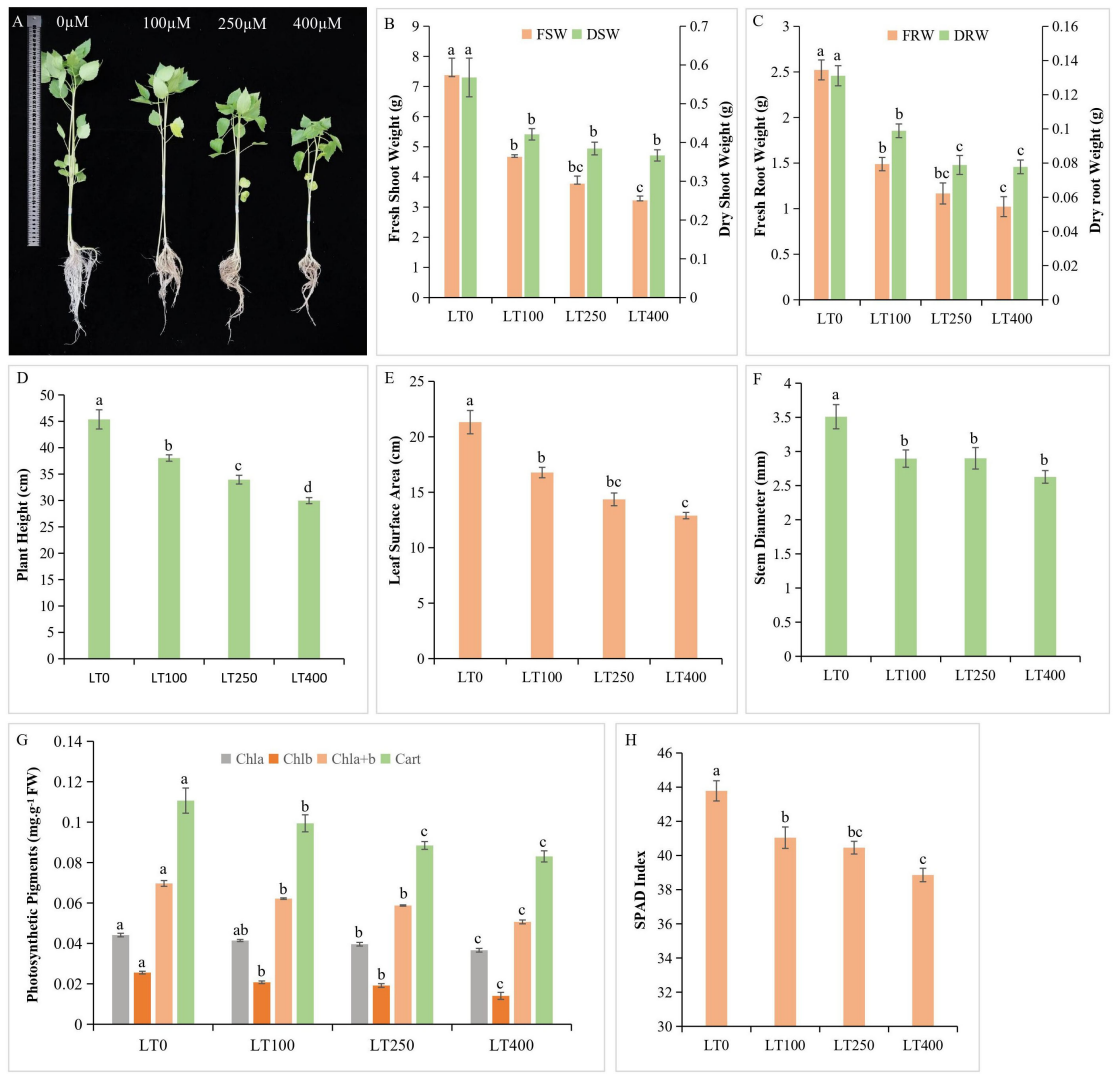
Kenaf growth characteristics under different concentrations of Cd. **(A)** Effect of different Cd concentrations on kenaf growth. **(B)** Fresh and dry shoot weight. **(C)** Fresh and dry root weight. **(D)** Plant height. **(E)** Leaf surface area. **(F)** Stem diameter. **(G)** Changes in photosynthetic pigment content and **(H)** SPAD index. The X-axis depicts different Cd concentrations as LT0, LT100, LT250, and LT400. The bars represent the mean standard error (n = 3). Significant differences between treatments at *p* ≤ 0.05 are indicated by different lowercase letters. SPAD, soil plant analysis development.

Cadmium exposure resulted in deleterious effects on kenaf, but the effects on growth were less in the low Cd treatment than in the high Cd treatment compared with control plants (**Figure 1A**). In kenaf, Cd treatment resulted in a significant decrease of 36.74%–56.35% in shoot FW and 40.96%–59.47% in root FW compared to control, whereas DW followed the same trend and decreased by 25.83%–35.42% in shoot and 24.57%–40.67% in roots (**Figures 1B, C**), indicating that roots were more adversely affected by Cd than shoots. With increasing Cd concentration, there was a corresponding decrease in height and LSA of the kenaf plant, with 400 μM Cd treatment resulting in the most significant decrease of 44.39% and 39.51% in plant height and LSA, respectively (**Figures 1D, E**), whereas Cd stress affected stem diameter compared to the control plants, although the differences between Cd treatments were not statistically significant (**Figure 1F**).

### 3.2 Changes in photosynthetic pigments under Cd stress

The levels of chlorophyll *a*, chlorophyll *b*, total chlorophyll, and carotenoids in plants exhibited a gradual decline of 4.54%–15.90%, 20%–44%, 10.14%–26.08%, and 10.90%–24.54%, respectively, as the Cd concentration increased compared to the control (**Figure 1G**). In addition, Cd exposure resulted in a significant decrease in the soil plant analysis development (SPAD) index, which is consistent with the results of photosynthetic pigment analysis (**Figure 1H**).

### 3.3 Cd accumulation and transport in kenaf

**Figure 2:**
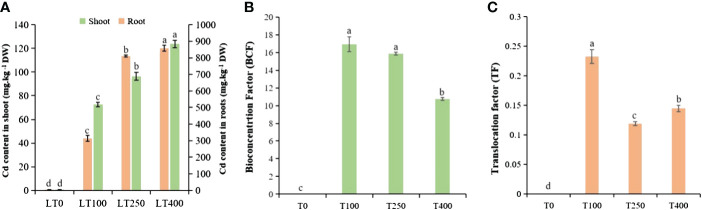
Cadmium accumulation and transport in kenaf under different concentrations of Cd stress. **(A)** Cd content in kenaf root and shoot tissues after exposure to various Cd concentrations. **(B)** BCF and **(C)** TF of Cd in kenaf. The X-axis depicts different Cd concentrations as LT0, LT100, LT250, and LT400. The bars represent the mean standard error (n = 3). Significant differences between treatments at *p* ≤ 0.05 are indicated by different lowercase letters. BCF, bioconcentration factor; TF, translocation factor.

The varying levels of Cd uptake under the different Cd concentrations indicated a concentration-dependent behavior of kenaf under Cd stress (**Figure 2A**). The Cd concentration in the shoots and roots of plants exposed to 100 μM Cd was 72.55 and 313.64 mg/kg DW, respectively; when treated with 400 μM Cd, the Cd content in the shoots and roots increased dramatically to 123.73 and 858.11 mg/kg DW, respectively. This represents an increase of approximately 70.54% and 173.59% in Cd concentrations in the shoots and roots of kenaf, respectively, compared to the control. The roots accumulated the most Cd followed by the shoots, indicating that the roots of kenaf plants serve as the primary site for Cd accumulation and storage (Cao et al., 2024). The BCF and TF of kenaf plants decreased with increasing Cd concentration, and low Cd treatment (100 μM) resulted in a higher BCF and TF than high Cd treatment (400 μM) (**Figures 2B, C**). These findings demonstrated that a high Cd concentration severely inhibited kenaf growth and development, resulting in decreased Cd enrichment and transport.

### 3.4 Cell wall polysaccharide component analysis

**Figure 3:**
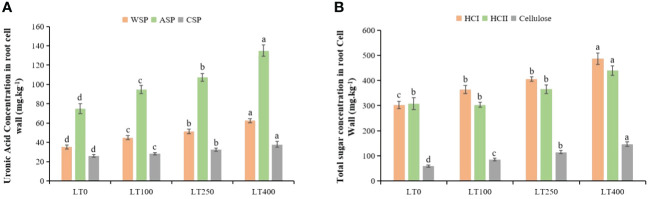
Effects of Cd exposure on the concentrations of kenaf cell wall polysaccharide components. **(A)** Uronic acid concentrations in water-soluble pectin (WSP), alkali-soluble pectin (ASP), and chelate-soluble pectin (CSP) in root tissues. **(B)** Total sugar concentrations in hemicellulose 1 (HCI), hemicellulose II (HCII), and cellulose in root tissues. The X-axis depicts different Cd concentrations as LT0, LT100, LT250, and LT400. The bars represent the mean standard error (n = 3). Significant differences between treatments at *p* ≤ 0.05 are indicated by different lowercase letters.

Cadmium stress increased the uronic acid concentration of all three types of pectins in the cell wall (**Figure 3A**). The uronic acid content of WSP, ASP, and CSP in the root cell wall exhibited an increase of 27.40%–77.30%, 26.62%–80.18%, and 7.97%–45.36%, respectively, when exposed to varying concentrations of Cd as compared to the control plants. In addition, Cd also induced HCI, HCII, and cellulose content accumulation in the root cell wall (**Figure 3B**), and their content substantially increased, particularly under high Cd treatment (400 μM Cd). Under varying Cd concentrations, the total sugar content of HCI, HCII, and cellulose in root cell walls increased by 20.62%–33.52%, 18.54%–20.41%, and 43.88%–72.43% respectively, compared to control plants.

### 3.5 Changes in oxidative stress indicators, antioxidative enzymes, and proline

**Figure 4:**
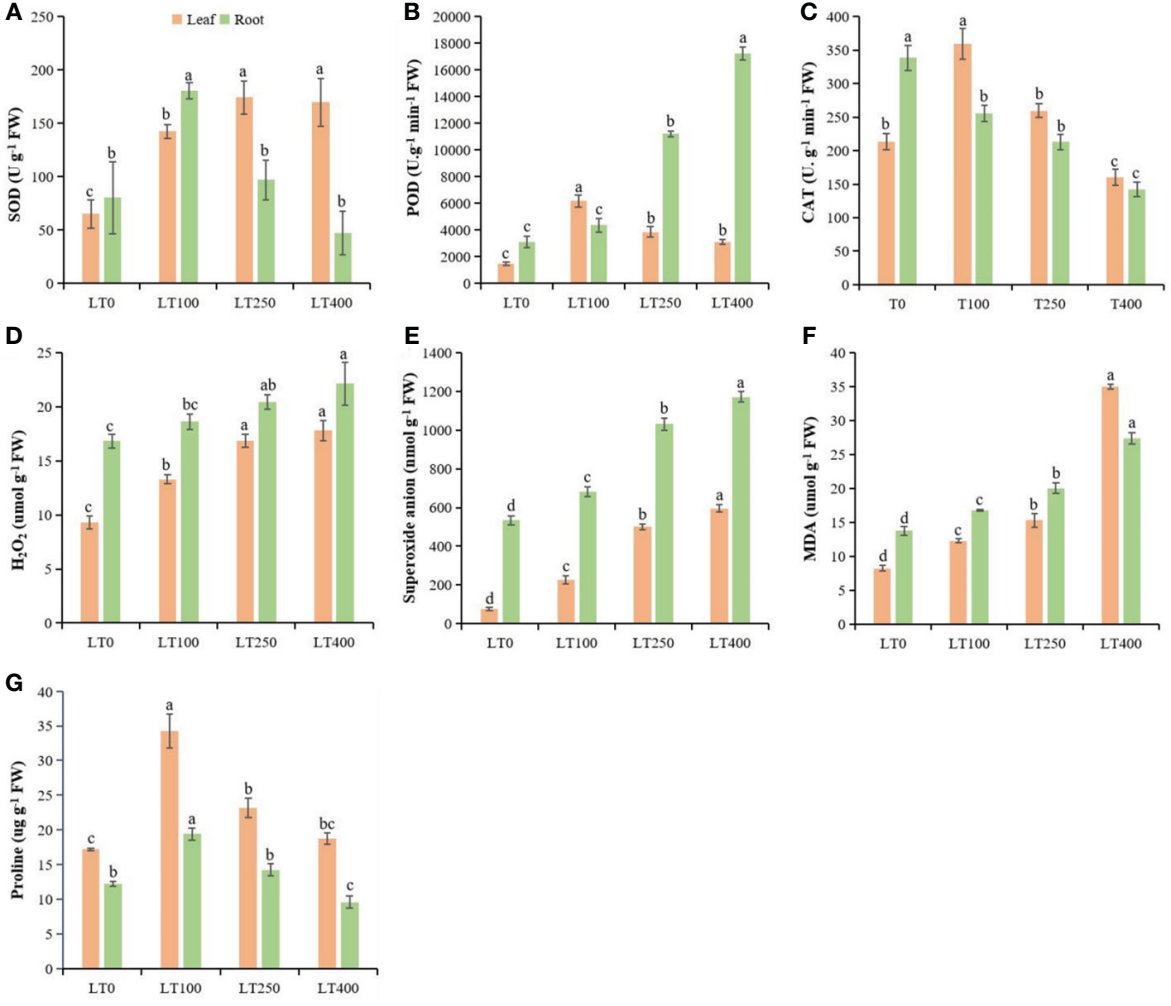
Physiological parameters in kenaf leaves and roots after 10 days of different Cd treatments. **(A–C)** SOD, POD, and CAT antioxidative enzyme activities, respectively. **(D–F)** Oxidative stress indicators H_2_O_2_ content, superoxide anion, and MDA content, respectively. **(G)** Protein content. The X-axis depicts different Cd concentrations as LT0, LT100, LT250, and LT400. The bars represent the mean standard error (n = 3). Significant differences between treatments at *p* ≤ 0.05 are indicated by different lowercase letters. SOD, superoxide dismutase; POD, peroxidase; CAT, catalase; MDA, malondialdehyde.

The SOD of kenaf leaves increased by 118.603%–160.46%, while POD and CAT activities were first increased and then began to decline with increasing Cd concentrations (**Figures 4A–C**). The SOD, POD, and CAT enzymes in kenaf roots followed a different pattern than in the leaves. In roots, SOD activity increased after 100 μM Cd treatment and then decreased and was significantly lower at the highest Cd concentration compared to the control (**Figure 4A**). POD activity increased by 40.59%–454.65%, but CAT activity decreased by 24.38%–57.98% in roots under different Cd treatments compared to the control (**Figures 4B, C**). The changes in Cd-induced oxidative stress indicators (H_2_O_2_, O_2_^−^, and MDA) in both roots and leaves of kenaf were increased at all tested Cd concentrations compared to the control (**Figures 4D–F**). The increase in H_2_O_2_, O_2_^−^, and MDA content was 42.38%–90.99%, 196.38%–680.76%, 49.03%–323.97%, respectively, in leaves and 10.57%–31.35%, 27.99%–119.32%, and 21.95%–98.69%, respectively, in roots under different Cd treatments compared to the control. In addition, the proline content initially increased and then declined following rising Cd concentration, and the largest increase in proline content was 98.83% in leaves and 57.99% in roots at 100 μM Cd treatment (**Figure 4G**). Furthermore, Cd stress had a significant effect on leaf proline content but a limited effect on root proline content of kenaf under varying Cd concentrations.

### 3.6 AsA–GSH cycle antioxidants

At all tested Cd concentrations, the GSH content in leaves declined by 27.43%–35.73%, whereas the GSSG level increased by 29.54%–193.34% (**Table 1**) compared to the control, resulting in a decreased GSH/GSSG ratio with increasing Cd concentration. In roots, the GSH content decreased 17.54%–34.85%, while the GSSG content increased at 100–250 μM Cd compared to the control and then decreased at 400 μM Cd, but it remained significantly greater than that of the control. Consequently, the value of the GSH/GSSG ratio decreased by 35.64%–68.29% under Cd treatments compared to the control. The AsA and DHA contents in leaves increased by 39.44%–170.61% and 20.54%–56.76%, respectively, under different Cd treatments compared to the control (**Table 1**). As a result, the AsA/DHA ratio rose as the Cd concentration increased. In roots, AsA content decreased (33.74%–47.66%), whereas DHA content increased (16.44%–37.61%) under varied Cd treatment compared to control, lowering the AsA/DHA ratio (**Table 1**).

**Table 1:** The contents of reduced ascorbate (AsA) and dehydroascorbate (DHA); ratios of AsA/DHA, reduced glutathione (GSH), and oxidized glutathione (GSSG); and ratios of GSH/GSSG in the leaves and roots of kenaf plants grown under varying Cd concentrations.

	Treatments	AsA	DHA	AsA/DHA ratio	GSH	GSSG	GSH/GSSG ratio
**Leaf**	LT0	765.67 ± 104.29 c	631.83 ± 67.50 b	1.21 ± 0.18 b	491.01 ± 36.84 a	89.97 ± 17.24 b	5.56 ± 0.96 a
	LT100	1,067.65 ± 92.15 c	729.35 ± 12.84 b	1.46 ± 0.17 b	356.3 ± 15.66 b	116.55 ± 26.14 b	3.16 ± 0.73 b
	LT250	1,559.19 ± 133.80 b	751.90 ± 21.74 ab	2.07 ± 0.15 a	383.55 ± 25.85 bc	241.2 ± 28.93 a	1.59 ± 0.09 c
	LT400	2,072.00 ± 213.09 a	940.43 ± 176.13 a	2.24 ± 0.39 a	315.57 ± 20.88 c	263.92 ± 29.80 a	1.20 ± 0.14 c
**Root**	LT0	1,991.64 ± 193.11 a	1,028.121 ± 123.91 b	1.95 ± 0.28 a	545.05 ± 46.11 a	87.24 ± 15.58 b	6.35 ± 0.91 a
	LT100	1,432.07 ± 20.51 b	1,197.24 ± 53.36 ab	1.10 ± 0.0.06 b	449.43 ± 15.78 b	113.70 ± 25.99 b	4.08 ± 0.84 b
	LT250	1,319.48 ± 45.14 b	1,367.43 ± 40.04 a	1.04 ± 0.04 b	452.46 ± 16.05 b	215.03 ± 16.83 a	2.11 ± 0.19 c
	LT400	1,342.32 ± 130.38 b	1,114.83 ± 47.42 b	1.20 ± 0.07 b	355.06 ± 35.53 c	176.42 ± 16.79 a	2.01 ± 0.05 c

The average of three biological replicates ± SD is used to present each value. Values showing different lowercase letters are significant as per Duncan’s least significant difference (LSD) test at p ≤ 0.05. LT indicates cultivar code, and numbers 0, 100, 250, and 400 denote the Cd treatment.

### 3.7 Normalization and basic analyses of metabolite data

A total of 678 metabolites (identified by combining positive and negative ions) were detected in all samples. Following data normalization, a hierarchical clustering heatmap of metabolites was constructed (**Supplementary Figure 1**), and then unsupervised PCA was performed in order to examine the overall distribution among samples and the extent of dispersion between groups. The findings demonstrated a notable distinction between the LT0 treatment group and the other two treatment groups (LT100 and LT400). The proximity of the data points between different treatment groups suggested a similarity in the composition and content of metabolites within the same samples (**Supplementary Figure 2A**). More than 52.9% and 28.1% variability were explained by PC1 and PC2, respectively, with PC1 accounting for the largest proportion. Subsequently, PLS-DA was conducted to identify global variations in metabolic profiles across different treatment groups. The PLS-DA score plot exhibited distinct clustering of sample points belonging to the same treatment, whereas sample points from different treatments demonstrated notable segregation (**Supplementary Figure 2B**). The PCA and PLS-DA findings demonstrated that the two models were capable of distinguishing between the three treatments. Additionally, they showed that the metabolite contents of kenaf significantly varied depending on the Cd treatments.

### 3.8 Differentially accumulated metabolites in response to Cd stress

**Figure 5:**
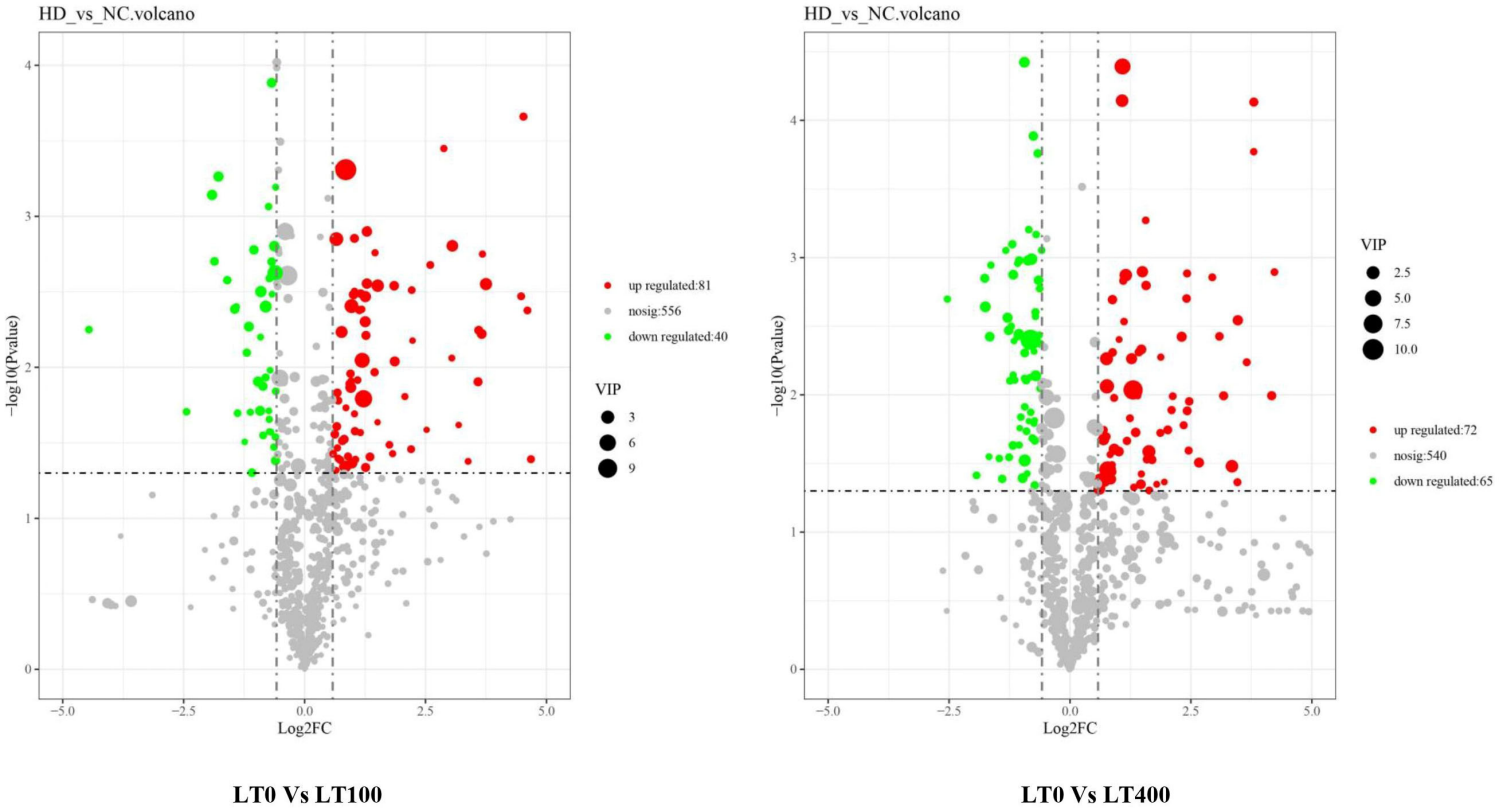
Volcano plot of Cd-induced differential metabolite in LT0 vs. LT100 and LT0 vs. LT400 comparison groups of kenaf leaves.

Univariate analysis with all metabolites found in both comparison groups was performed using both fold change (FC) and *t*-test criteria (FC > 1.5, or metabolites with FC < 0.67 and *p*-value <0.05). The differential metabolites determined by univariate statistical analysis were visualized through the volcano plot in **Figure 5**. The results showed that the abundance of 121 and 135 metabolites was significantly different in the LT0 vs. LT100 and LT0 vs. LT400 comparison groups, respectively. In the comparison group of LT0 vs. LT100, 81 metabolites exhibited upregulation, while 40 metabolites showed downregulation. Similarly, in the comparison group of LT0 vs. LT400, 72 metabolites were upregulated and 65 metabolites were downregulated. Additional screening was conducted by calculating the VIP using a *t*-test combined with PLS-DA (VIP > 1, *p* ≤ 0.05) to screen potential marker metabolites. A total of 38 differential metabolites from the LT0 vs. LT100 comparison group and 37 metabolites from the LT0 vs. LT400 comparison group (**Supplementary File 1**) were identified, and a hierarchical clustering heatmap was constructed to evaluate a more comprehensive and intuitive display of the differences in the expression patterns of metabolites under different Cd treatments (**Supplementary Figure 3**). The hierarchical clustering results clearly demonstrated that Cd stress altered the metabolic profile of kenaf leaves in comparison to the control, which reflected the aforementioned metabolite changes.

**Figure 6:**
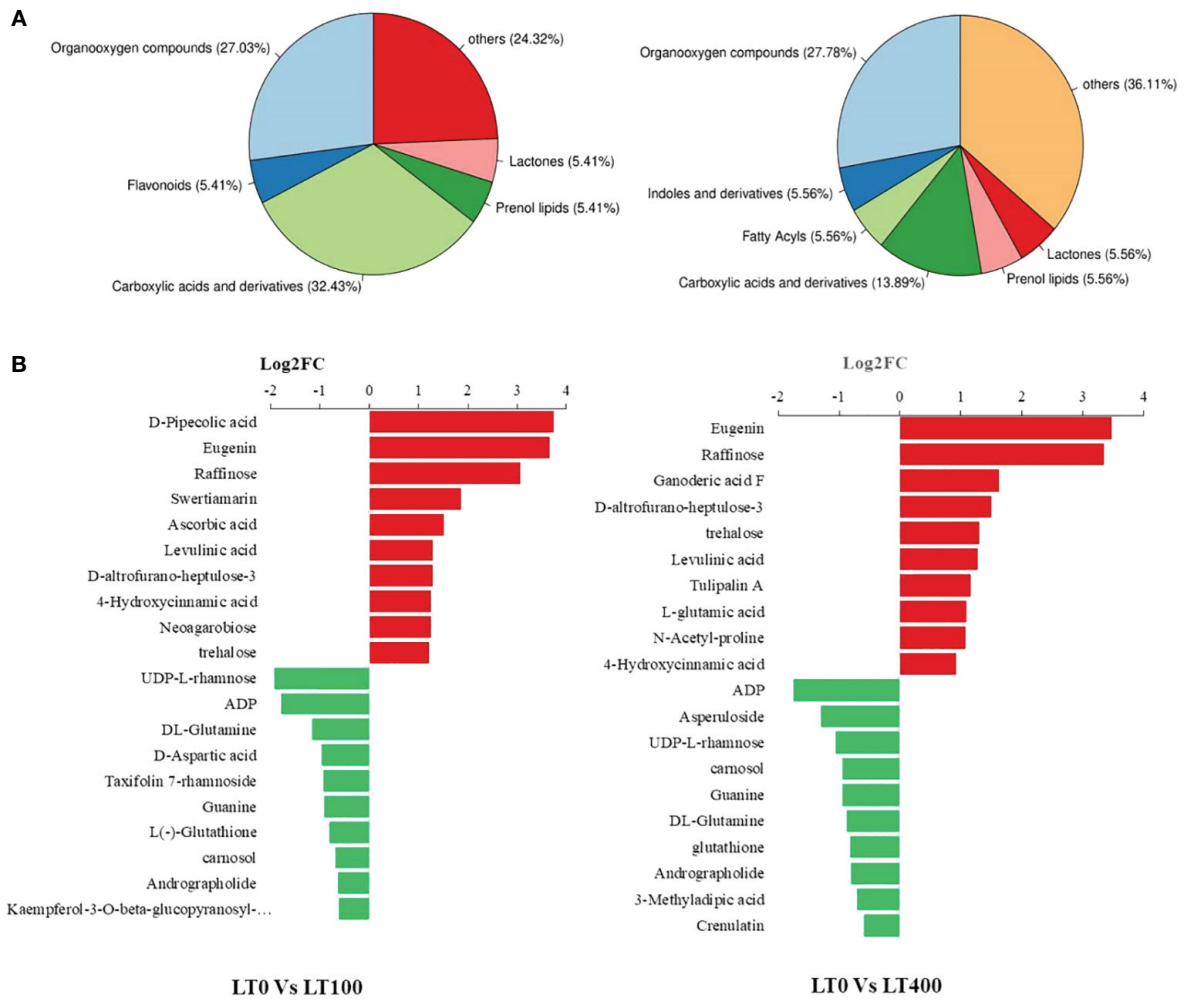
Classification of differentially accumulated metabolites in kenaf leaves. **(A)** Classification of differential metabolites in LT0 vs. LT100 and LT0 vs. LT400 comparison group. **(B)** Up- and downregulation of the top 10 differential metabolites in LT0 vs. LT100 and LT0 vs. LT400 comparison group.

We classified these metabolites into six major groups in the LT0 vs. LT100 comparison group, with carboxylic acid and derivatives (32.43%) being the most prevalent metabolites followed by organooxygen compounds (27.03%). In the LT0 vs. LT400 comparison group, differential metabolites were divided into seven major groups with organooxygen compounds having the highest abundance (27.78%), followed by carboxylic acid and derivatives (13.89%) (**Figure 6A**). Moreover, we identified the top 10 upregulated and downregulated differential metabolites in each comparison group based on the difference in FC in metabolite accumulation (**Figure 6B**). The results revealed that d-pipecolic acid was the most significantly increased and UDP-l-rhamnose was the most significantly decreased differential metabolite in the LT0 vs. LT100 comparison group, while Eugenie and adenosine-5′-diphosphate were the least significant increased and decreased differential metabolites in the LT0 vs. LT400 comparison group, respectively. Through further screening, we discovered 22 metabolites that shared accumulation across the two comparison groups (**Supplementary File 1**), indicating variations in the metabolic response to Cd stress. Among them, most compounds belonging to amino acids and derivatives (*N*-acetyl-proline, l-glutamic acid, and levulinic acid), carbohydrates (gluconic acid, Palatinose, d-altrofurano-heptulose-3, raffinose, and trehalose), and terpenoids (ganoderic acid F) showed increased accumulation, while major compounds belonging to alcohols and polyols (3-*O*-caffeoylquinic acid and cryptochlorogenic acid), flavonoids (kaempferol-3-*O*-beta-glucopyranosyl-7-*O*-alpha-rhamnopyranoside), and nucleotides (adenosine-5′-diphosphate, guanine, and UDP-l-rhamnose) were decreased.

### 3.9 Annotation and enrichment analysis of metabolites in KEGG database

**Figure 7:**
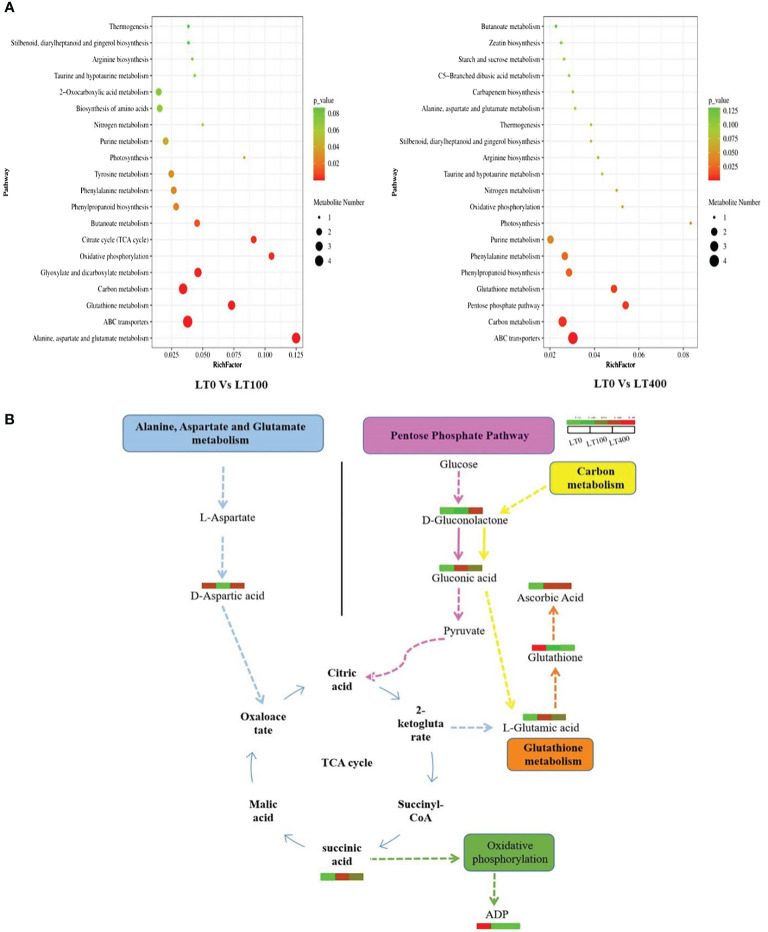
KEGG annotation of differentially accumulated metabolites. **(A)** List of the top 20 KEGG pathways in LT0 vs. LT100 and LT0 vs. LT400 comparison groups in which differential metabolites were significantly upregulated or downregulated. Rich factor is displayed along the X-axis, and pathway names are along the Y-axis. The number of differential metabolites is shown by the size of the bubbles. The pathway enrichment level is represented by the bubble color. **(B)** Metabolite changes in significantly enriched metabolic pathways in kenaf after 10 days of Cd stress. KEGG, Kyoto Encyclopedia of Genes and Genomes.

The pathways of the differential metabolites were annotated using the KEGG database, and the metabolites were categorized based on the associated pathways or their respective functions. The distribution of KEGG pathways was shown at the second hierarchical stage (**Supplementary Figure 4**). The largest pathway group was the global and overview map followed by carbohydrate metabolism in both comparison groups. The enrichment analysis results revealed that the differential metabolites in the comparison group of LT0 vs. LT100 exhibited significant enrichment in alanine, aspartate, and glutamate metabolism, oxidation phosphorylation, and citrate (TCA) cycle, while ABC transporter, carbon metabolism, pentose phosphate pathway, and glutathione metabolism were the most enriched pathways in the LT0 vs. LT400 comparison group (**Figure 7A**). A schematic representation of the metabolic network of the differential metabolites, which were shown to be distinct among the comparison groups, is presented by integrating the identified differential metabolites and enriched pathways (**Figure 7B**).

### 3.10 Gene expression analysis

**Figure 8:**
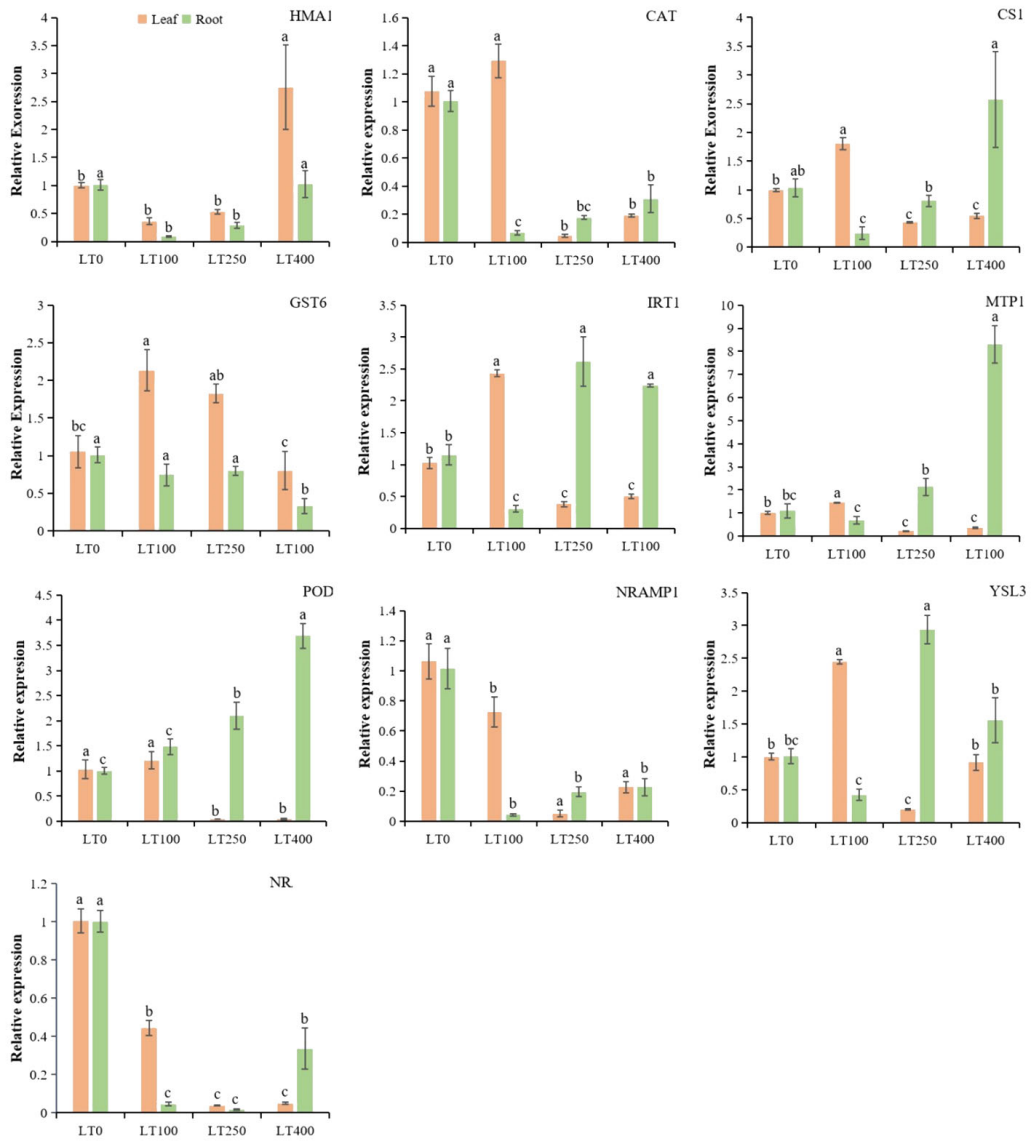
Relative expression levels of known divalent heavy metal ion transporter genes, Cd-resistance genes, and antioxidant enzyme genes in roots and leaves of kenaf under different Cd treatments. The X-axis depicts different Cd concentrations as LT0, LT100, LT250, and LT400. The bars represent the mean standard error (n = 3). Significant differences between treatments at *p* ≤ 0.05 are indicated by different lowercase letters.

The gene expression profiles of Cd-inducible transporter genes, heavy metal ions transporter genes, Cd-resistance genes, and antioxidant enzyme genes showed that these genes had tissue-specific expression patterns under different Cd treatments (**Figure 8**). The expression levels of cysteine synthase 1 (*CS1*), *CAT*, iron-regulated transporter-1 (*IRT1*), metal tolerance protein (*MTP1*), *POD*, and yellow stripe like-3 (*YSL3*) were higher only at 100 μM; glutathione *S*-transferase-6 (*GST6*) was highly expressed at 100 and 250 μM in leaves. Heavy metal atpase-1 (*HMA1*) gene was only expressed in leaves under 400 μM Cd concentration. Natural resistance-associated macrophage-1 (*NRAMP1*) and *NRAMP6* were downregulated in leaves, indicating their negative correlation between Cd concentrations and these gene expressions. However, *IRT1*, *MTP1*, *POD*, and *YSL3* were specifically expressed in the root at 250 μM and 400 μM, while *CS1* was highly expressed in the root only under 400 μM Cd concentration. In addition, *NRAMP1*, *NRAMP6*, *HMA1*, *CAT*, and *GST6* were downregulated in roots at all tested Cd concentrations. These findings suggested that these genes might have distinct functions in the relevant tissues under different Cd concentrations.

## 4 Discussion

The primary cause of plant growth retardation and biomass loss is Cd toxicity to the photosynthetic process. The rate of photosynthesis reflects plant growth performance, which in turn is proportional to its chlorophyll and carotenoid content (Anwaar et al., 2015). The current study revealed that Cd exposure resulted in a decrease in the content of chlorophyll *a*, chlorophyll *b*, total chlorophyll, carotenoid, and SPAD index in kenaf, with an increase in Cd concentrations (**Figures 1G, H**), leading to a significant alteration in the net growth of plant height as well as plant fresh and dry biomass (**Figures 1B–D**). Consistent with our results, other scientists have also found that Cd stress can inhibit photosynthetic pigments in *Sassafras* seedlings (Zhao et al., 2021), maize (Anjum et al., 2016b), and *Capsicum annuum* (Hasan et al., 2022) and that the damage to photosynthetic pigments is significantly correlated with the level of heavy metal stress. A possible explanation for the decrease in chlorophyll content could be the interference of Cd with magnesium (Mg) and iron (Fe) sources necessary for the synthesis process (Van Assche and Clijsters, 1990). The substitution of the Mg atom in chlorophyll with Cd results in the formation of the chlorophyll–Cd complex. According to Siedlecka et al. (1998), the replacement negatively impacts the photosynthetic apparatus. Furthermore, the reduction in chlorophyll content under Cd stress can be attributed to the inhibition of enzyme synthesis involved in chlorophyll biosyntheses, such as d-aminolevulinic acid and protochlorophyllide reductase (Huang et al., 1997; Gonçalves et al., 2009) as well as the increased degradation of these enzymes (Somashekaraiah et al., 1992).

The differential rates of Cd absorption by roots and translocation from roots to shoots explain the different Cd-resistance levels of kenaf. Our study revealed that Cd is primarily accumulated in the roots, followed by the shoots (**Figure 2A**). Plants can withstand Cd by vacuole compartmentalization, Cd chelation facilitated by cytoplasmic organic acids or peptides, and Cd chelation by cell wall components (Deng et al., 2019; Wang et al., 2022). The cell wall acts as the first line of defense against Cd stress (Richter et al., 2017). It is mostly composed of proteins and polysaccharides, which contain negatively charged sites that prevent Cd from entering cells through the cytoplasmic membrane (Liu et al., 2015). Previous studies have demonstrated increased pectin, cellulose, and hemicellulose levels in plant cell walls under Cd stress, which serve as the primary site of preferential Cd binding in *Solanum lycopersicum* (Jia et al., 2019) and *Arabidopsis halleri* (Meyer et al., 2015). The levels of kenaf root cell wall components increased at all concentrations of Cd compared to the control group, which is consistent with the studies mentioned above. Through the analysis of cell wall components, cell wall synthesis, and cell wall-modifying enzyme-related genes in tomatoes, Jia et al. (2019) also found that Cd binding to the cell wall significantly increased as the concentration of cell wall components increased. In the present study, the high Cd concentration in the root tissue of kenaf highlights the importance of Cd chelation within the cell wall as the concentration of cell wall components increases and serves as a vital and efficient mechanism for Cd tolerance in kenaf. This approach could further attenuate the transport of Cd from roots to above-ground plant parts, as shown by the observed decrease in Cd concentrations in the aerial parts of the plant (**Figure 2A**). However, in our future research, we need to distinguish and analyze Cd compartmentalization between the cell wall, cytoplasm, and vacuole.

For the goal of phytoremediation, BCF and TF are direct methods to evaluate the ability of a plant to accumulate and translocate heavy metals (Yan et al., 2022). In this study, the kenaf plant showed declining trends in BCF and TF with increasing Cd concentrations, and low Cd concentrations could lead to higher Cd accumulation in roots and translocation from roots to above-ground parts when compared to high Cd concentrations (**Figures 2B, C**). Thus, our research indicated that kenaf is more effective as a remediation candidate at low Cd concentrations than at high Cd concentrations. Plants exposed to environmental stress, either individually or in combination, result in oxidative stress, poor redox equilibrium, and an excess accumulation of ROS (Hasanuzzaman et al., 2020). In this study, Cd-treated kenaf plants produced high ROS (H_2_O_2_ and O_2_^−^) and MDA content in leaf and root tissues (**Figures 4D–F**), indicating substantial lipid peroxidation and membrane damage in Cd-treated kenaf plants, which is comparable with *Oryza sativa* (Bhuyan et al., 2020), *Withania somnifera* (Mishra et al., 2019), and Brassica juncea (Al Mahmud et al., 2018) under Cd stress, and it was believed that ROS accumulation caused by high Cd toxicity exceeded the damage threshold over time and compromised the integrity of the cell membrane. Antioxidative enzymes (SOD, POD, and CAT) play a crucial role in enabling plants to withstand oxidative stress by effectively neutralizing ROS generated as a result of abiotic stressors (Anjum et al., 2015). SOD enzyme converts O_2_^−^ into H_2_O_2_, which is then broken down into water and molecular oxygen by CAT and POD antioxidative enzymes (Sirhindi et al., 2015; Tanveer and Shabala, 2018). In our study, SOD activity increased, while POD and CAT activities decreased with increasing Cd concentrations in kenaf leaves (**Figures 4A–C**). Conversely, the activity of SOD in roots first increased and then decreased with increasing Cd treatment, whereas the trends for POD and CAT were decreasing and increasing, respectively. These results indicated that antioxidative enzymes in roots and leaves respond differently to Cd and suggested that Cd may become inhibitory above a given concentration, depending on the enzyme examined. Moreover, CAT activity, in addition to its detoxification roles, would also be a sensitive target of Cd toxicity in plants, which explained the negative correlation between Cd concentration and CAT activity, while increased SOD activity in leaves and POD activity in roots is intended to maintain the equilibrium of free radicals in plants and to improve kenaf tolerance to Cd stress. Proline is a component of compatible organic osmolytes (Anjum et al., 2016a), and the fact that proline content increased only at low Cd concentrations (**Figure 4G**) suggests that low Cd stress led to proline synthesis in plants to protect them from osmotic stress. This hardened the kenaf plants and made them tolerant to Cd toxicity.

The AsA–GSH cycle contributed to the efficient elimination of ROS through the action of SOD and CAT. The concentrations of AsA and GSH, along with their redox ratios, play a crucial role in governing plant physiology in response to diverse abiotic stress conditions (Jung et al., 2019). Ascorbate is an effective ROS scavenger used by APX to convert H_2_O_2_ to H_2_O (Gill et al., 2015). Under Cd stress, the AsA level and AsA/DHA ratio greatly increased in kenaf leaves, and a corresponding rise in the level of DHA is likely associated with enhanced glutathione-dependent DHAR activity and/or increased AsA synthesis. However, a decreasing trend in the AsA/DHA ratio was shown in roots under Cd stress. More research is needed to understand the distinct responses of AsA and DHA in the leaves and roots of kenaf plants. In addition, a decrease in the GSH pool under Cd stress suggests an increased oxidation of GSH to GSSG, formation of Cd–GSH complexes, or the biosynthesis of phytochelatins (PCs), which serve as the antioxidant defense mechanisms. It is well known that the GSH pool and the GSH/GSSG ratio are essential for preserving the cell’s redox state. The current study found a significant decrease in both the GSH pool and the GSH/GSSG ratio, indicating a negative correlation between the GSH pool and the alleviation of Cd-induced damage in kenaf. This is consistent with the findings obtained for *Vigna radiata* (Anjum et al., 2008, Anjum et al, 2011) and *Camellia sinensis* (Wang et al., 2023).

To conduct a more comprehensive investigation of the resistance mechanism of kenaf plants to Cd stress, the metabolites of kenaf leaves were studied. It was intriguing to discover that the differential metabolites in kenaf leaves under Cd stress between LT0 vs. LT100 and LT0 vs. LT400 were mainly enriched in amino acids and carbohydrates such as succinic acid, citric acid, l-glutamic acid, d-aspartic acid, delta-gluconolactone, ascorbic acid, and gluconic acid, which are important in plant stress resistance to metal toxicity. These differential metabolites are widely distributed in secondary metabolite synthesis pathways such as alanine, aspartate, and glutamate metabolism, TCA cycle, pentose phosphate pathway, and glutathione metabolism, implying that the priority of different pathways under low and high Cd stress influences the resistance mechanism in kenaf (**Figure 7B**). Succinic acid and citric acid are intermediates of the Krebs cycle that are essential for energy production and regulation of the mitochondrial TCA cycle (Tahjib-Ul-Arif et al., 2021). In this study, succinic acid and citric acid levels were found to be significantly higher in low Cd-treated plants, indicating increased resistance to Cd stress. This may be attributed to the plant’s efficient TCA cycle, which generates more energy in Cd-stressed conditions.

Glutathione and ascorbic acid are important components of the cellular antioxidant defense mechanism that regulates ROS and is involved in the sequestration of heavy metals (Dewhirst and Fry, 2018). Ascorbic acid content increased in both Cd treatments compared to LT0, whereas glutathione content decreased, consistent with physiological observations indicating that ascorbate plays an important role in enhancing kenaf resistance to Cd stress. Amino acids efficiently form complexes with metal ions in the cytoplasm, hence mitigating the adverse impacts of heavy metals on plants (Singh et al., 2016). Glutamic acid belongs to the α-ketoglutarate group of amino acids and is required for various metabolic processes, including alanine, aspartate, and glutamate metabolism, glutathione metabolism, and carbon metabolism. As a precursor of proline and arginine, glutamic acid provides a link between amino acids and respiratory metabolism (Ali et al., 2019). Previously, increased accumulation of glutamic acid was reported in water and salt stress *Glycine max* and *Sporobolus stapfianus*, respectively (Martinelli et al., 2007; Farhangi-Abriz and Ghassemi-Golezani, 2016). In this study, proline content increased only in the low Cd treatment group compared to LT0, which could be related to the promotion of glutamic acid that occurred only in the low Cd treatment group. Aspartic acid, a member of the oxaloacetate family of amino acids, is also differentially accumulated in response to Cd stress. This amino acid is essential to nitrogen and carbon metabolism, thereby participating in various metabolic pathways including the synthesis of other amino acids, nucleotides, and organic acids within the TCA cycle, as well as the production of sugars in glycolysis and hormones, all of which are indispensable for the growth and stress tolerance of plants (Ali et al., 2019).

The relative expression of randomly selected genes was measured to enhance comprehension of the uptake and tolerance mechanisms of kenaf under different Cd treatments (**Figure 8**). Multiple studies have observed an increase in H_2_O_2_ and MDA levels following exposure to Cd stress. Additionally, the levels of antioxidants such as SOD, POD, CAT, GSH, and GST were also found to increase (Fu et al., 2019), indicating the activation of the antioxidant system. Consistent with the aforementioned physiological findings, our data revealed that the expression of genes responsible for producing antioxidants, such as *POD*, *CAT*, and *GST6*, was differentially stimulated under the influence of Cd-induced stress in kenaf roots and leaves. However, opposite expression trends, concentrations, and tissue-specific antioxidant families were detected between root and shoot tissues, indicating that the Cd-induced stress response was distinct between tissues, as found by Fu et al. (2019) in *Brassica napus* and Wang et al. (2020) in *O. sativa*. Among the transporter genes, *IRT1* and *YSL3* genes are mainly involved in Cd uptake and transport in plants (Tao and Lu, 2022). Heterologous expression in yeast revealed that *OsIRT1* and *OsIRT2* are crucial transporters for Fe and Cd uptake (Jiang et al., 2021). Rice and *Arabidopsis* were more sensitive to Zn and Cd when *IRT1* was overexpressed (Jiang et al., 2021). Moreover, YSLs are transporters localized in the plasma membrane that are involved in the long-distance transport of metal ions from the root to the shoot (Verbruggen et al., 2009). Under Cd stress, the Cd translocation ratio was increased in *Arabidopsis* when *MsYSL1* or *SnYSL3* were overexpressed (Chen et al., 2018). In addition, plant MTPs belong to the cation diffusion facilitator (CDF) protein family. *MTP1*, a Zn^2+^/H^+^ vacuolar transporter, regulates Cd flow in *Thlaspi goesingense* (Kim et al., 2004). We found that *IRT1* and *MTP1* gene overexpression at 250 and 400 μM Cd can increase root Cd accumulation, while *YSL3* gene upregulation in kenaf roots can increase Cd transport at these concentrations. Within the leaves, the genes *IRT1*, *YSL3*, and *MTP1* were only found to be more active when exposed to a concentration of 100 μM. This indicates that these genes may serve different functions in leaves compared to roots when exposed to different levels of Cd. Furthermore, they may help reduce the accumulation of Cd in kenaf leaves by reducing the expression of these Cd transporters, as evidenced by the low TF observed under higher Cd stress. NRAMPs are major manganese (Mn) transporters that are also capable of transporting Zn, Mn, Fe, Cd, and As (Liu et al., 2022). However, given the reduced expression of *NRAMP1* and *NRAMP6* in the root and leaf of kenaf, it is unlikely that they are actively involved in Cd accumulation and transport in this plant.

## 5 Conclusion

**Figure 9:**
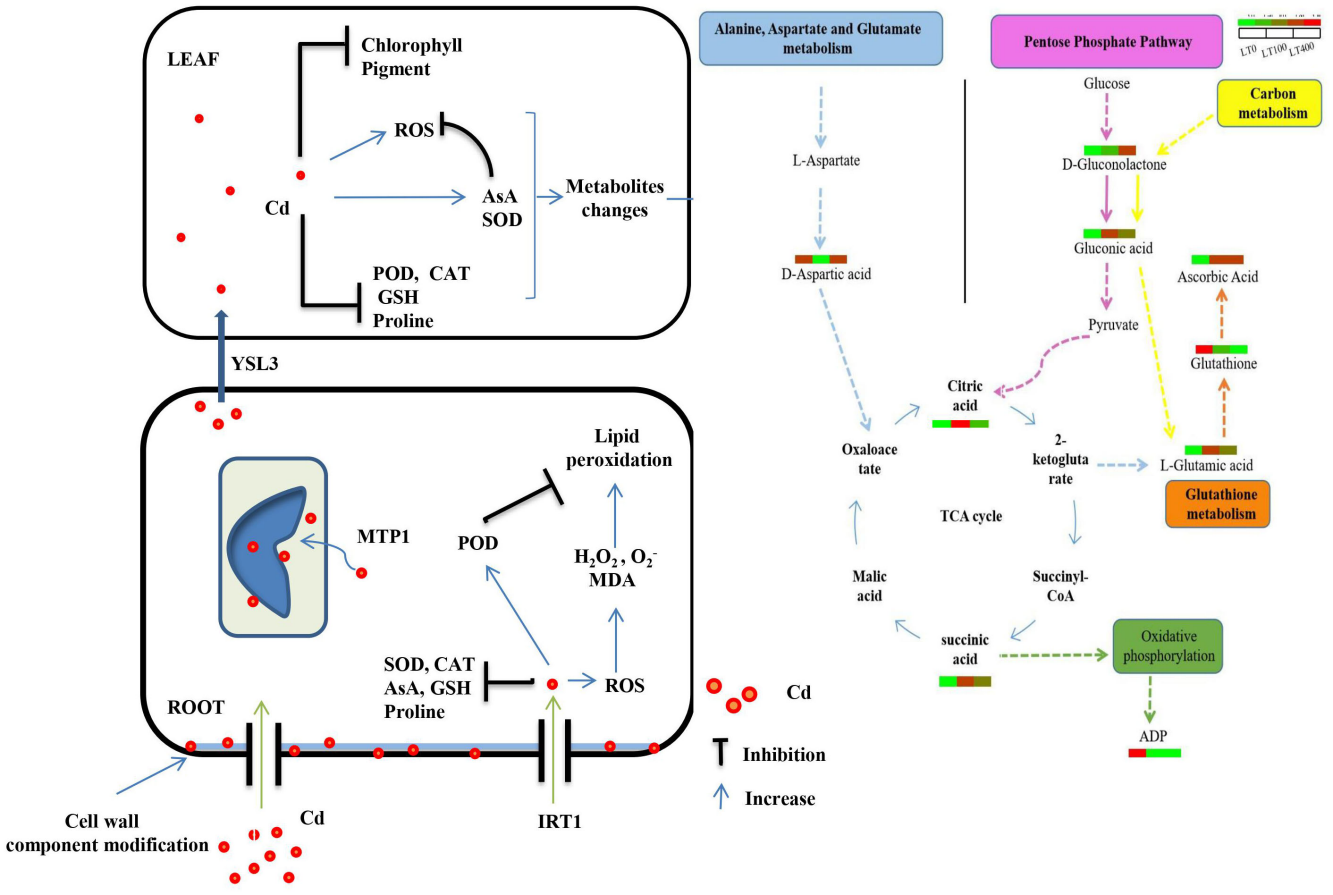
Speculations of toxicity, tolerance, and detoxification mechanism of Cd in kenaf.

In conclusion, **Figure 9** depicts a proposed process of Cd toxicity, tolerance, and detoxification in kenaf. Kenaf exhibited potential remediation ability at low Cd concentrations as indicated by biomass production, physiological indices, and metabolite accumulation. In addition, metabolite analysis revealed that low Cd concentrations (100 μM) regulated alanine, aspartate, and glutamate metabolism, oxidative phosphorylation, and the citrate (TCA) cycle, particularly by upregulating glutamic acid content, which maintained high proline content and increased kenaf tolerance to low Cd stress. ABC transporter, carbon metabolism, the pentose phosphate pathway, and glutathione metabolism were the most enriched pathways in the presence of high Cd concentrations (400 μM). Moreover, the increased expression of *IRT1* and *MTP1* genes and the high content of cell wall polysaccharide components in roots contributed to an increase in Cd accumulation in roots.

## Data availability statement

The original contributions presented in the study are included in the article/**Supplementary Material**. Further inquiries can be directed to the corresponding authors.

## Author contributions

WS: Data curation, Formal Analysis, Investigation, Methodology, Writing – original draft. SM: Data curation, Investigation, Writing – review & editing. JP: Methodology, Resources, Writing – review & editing. MR: Writing – review & editing. WQF: Investigation, Methodology, Writing – review & editing. DJL: Writing – review & editing. PWL: Funding acquisition, Resources, Writing – review & editing. YL: Funding acquisition, Resources, Writing – review & editing. PC: Conceptualization, Funding acquisition, Resources, Supervision, Writing – review & editing.
