# Supplementary material for: Integrated physiological and metabolomic responses reveal mechanisms of Cd tolerance and detoxification in kenaf (Hibiscus cannabinus L.) under Cd stress
Source: Front Plant Sci. 2024 Aug 8;15:1332426. doi: 10.3389/fpls.2024.1332426 (PMC11340530; doi:10.3389/fpls.2024.1332426)
Supplement: Supplementary file 1 [file DataSheet_1.docx]

**Integrated physiological and metabolomic responses reveal mechanisms of Cd tolerance and detoxification in kenaf (*Hibiscus cannabinus* L.) under Cd stress**

## Wajid Saeed^a^, Samavia Mubeen^a^, Jiao Pan^a^, Muzammal Rehman^a^, [Wangqiang Fang](https://papers.ssrn.com/sol3/cf_dev/AbsByAuth.cfm?per_id=5559333)^a^, [Dengjie Luo](https://papers.ssrn.com/sol3/cf_dev/AbsByAuth.cfm?per_id=5559331)^a^, Pingwu Liu ^b^, Yun Li^a^*, Peng Chen ^a^*

^a^ Guangxi Key Laboratory of Agro-environment and Agric-products Safety, Key Laboratory of Plant Genetics and Breeding, College of Agriculture, Guangxi University, Nanning 530004, PR China

^b^ Sanya Nanfan Research Institute of Hainan University, Sanya 572025, China

**Figure S1**


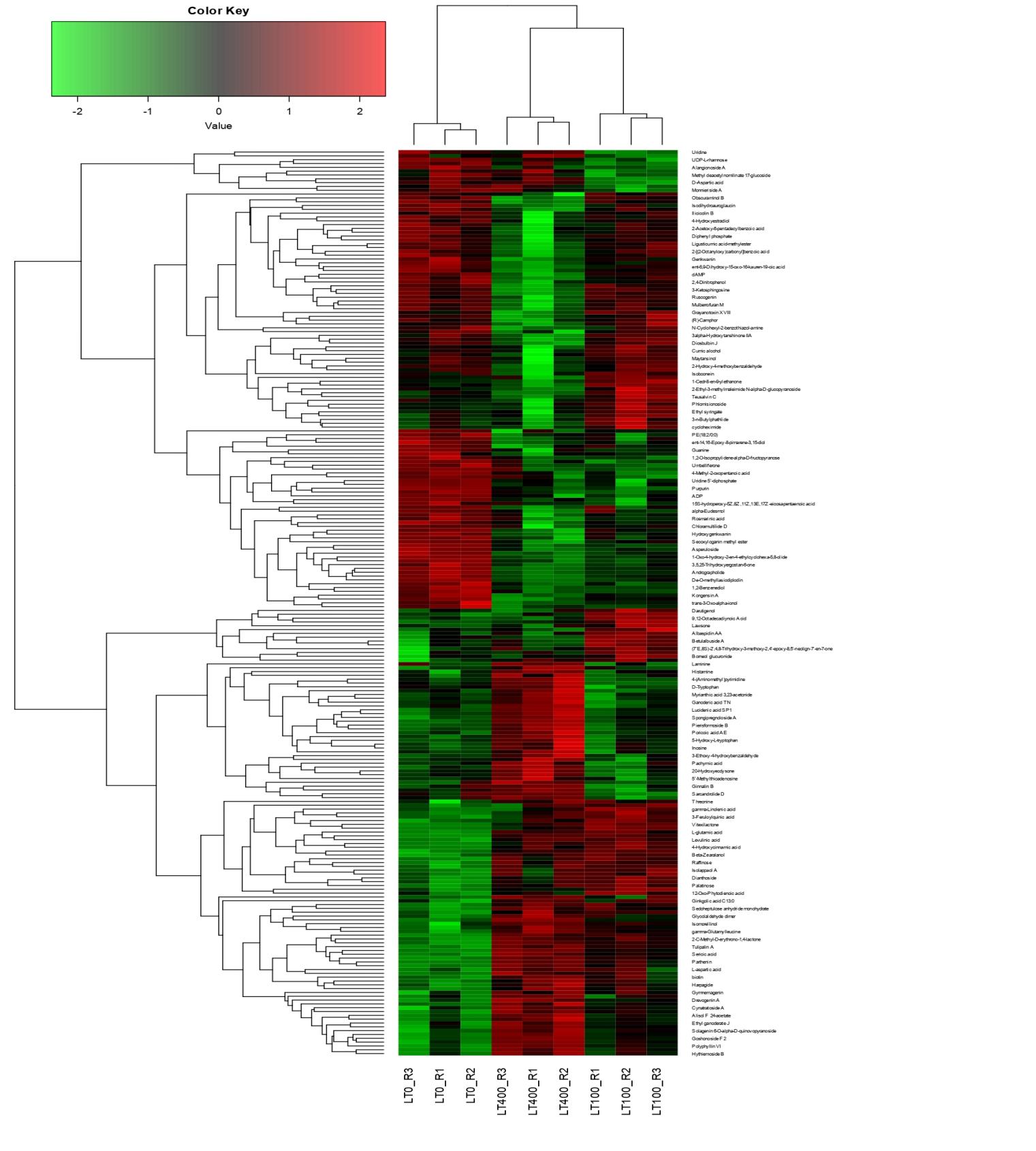


**Fig S1.** Heatmap showing the results of the clustering analysis of the total metabolites identified by combining positive and negative ions

**Figure S2**

***
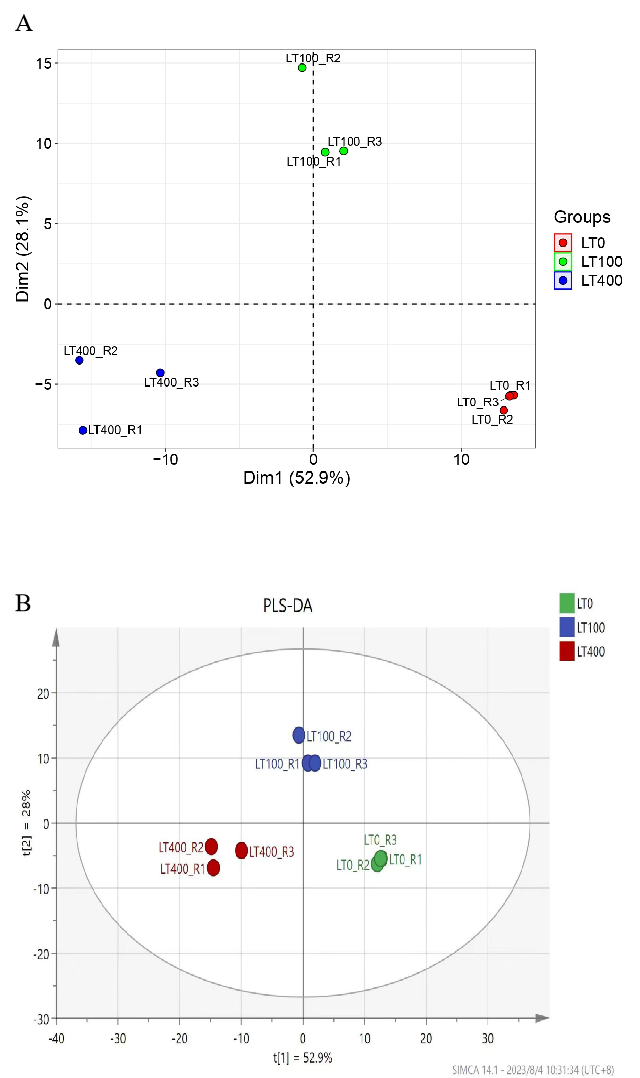
***

**Fig S2.** Quality control of metabolomics data. (A) PCA plot and (B) PLS-DA plot of metablomic data identified in both positive and negative ion mode.

**Figure S3**


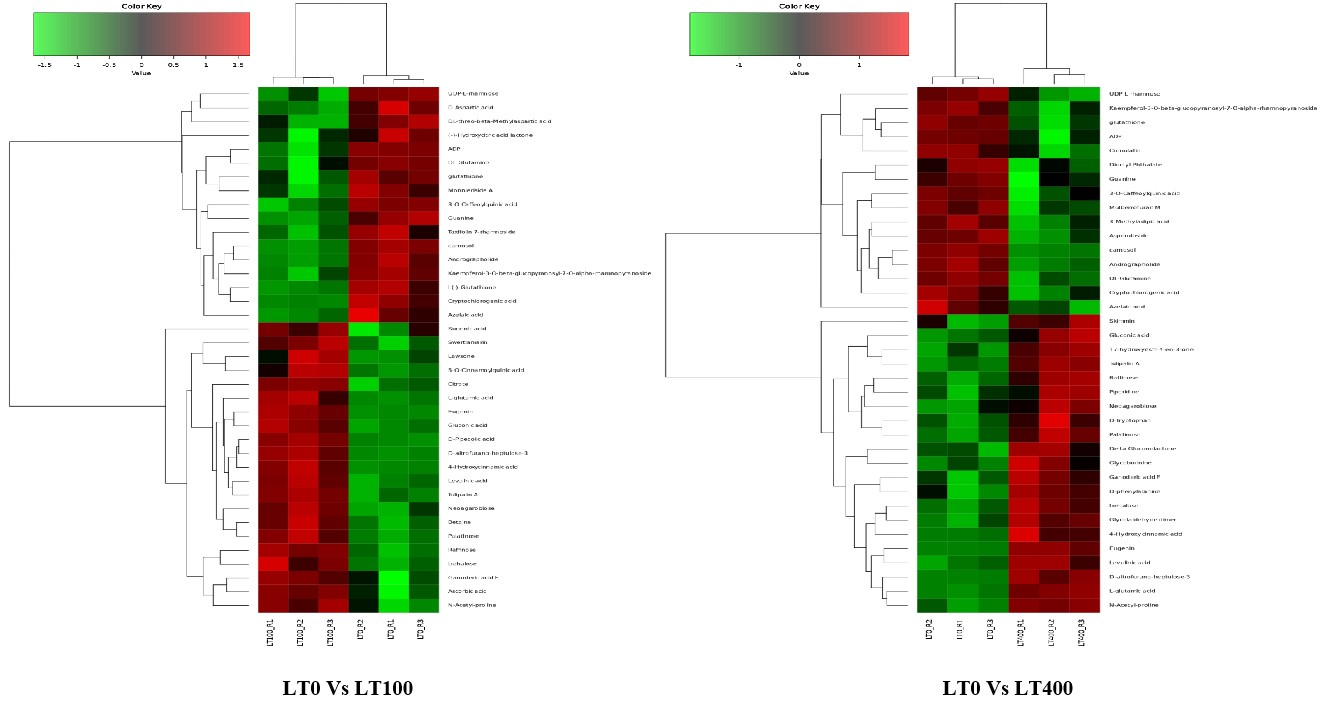


**Fig S3.** Hierarchical clustering heatmap showing the up- and downregulated differential metabolites.

**Figure S4**


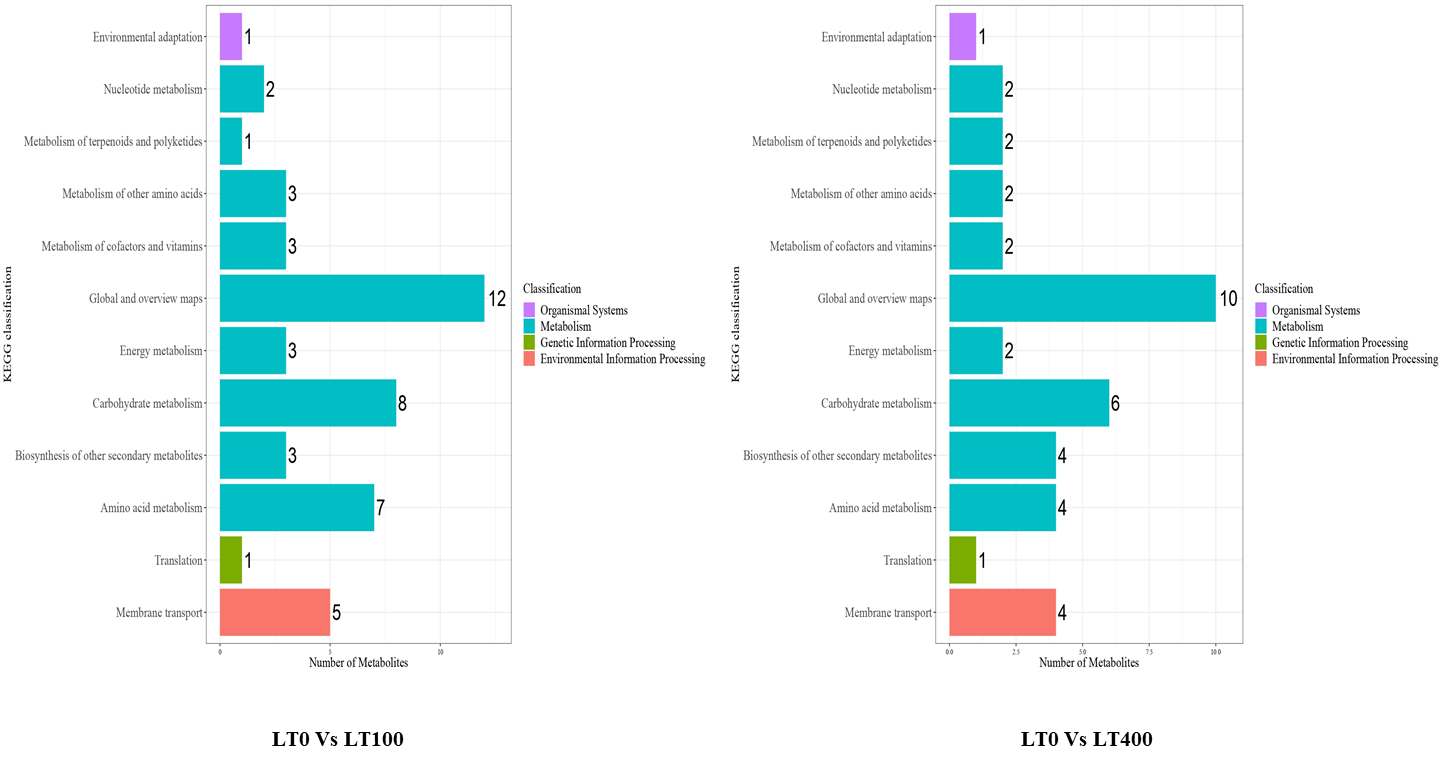


**Fig S4.** Hierarchical classification of KEGG metabolic pathways
