# Supplementary material for: Integrated physiological and metabolomic responses reveal mechanisms of Cd tolerance and detoxification in kenaf (Hibiscus cannabinus L.) under Cd stress
Source: Front Plant Sci. 2024 Aug 8;15:1332426. doi: 10.3389/fpls.2024.1332426 (PMC11340530; doi:10.3389/fpls.2024.1332426)
Supplement: Supplementary file 3 [file DataSheet_3.docx]

**Integrated physiological and metabolomic responses reveal mechanisms of Cd tolerance and detoxification in kenaf (*Hibiscus cannabinus* L.) under Cd stress**

## Wajid Saeed^a^, Samavia Mubeen^a^, Jiao Pan^a^, Muzammal Rehman^a^, [Wangqiang Fang](https://papers.ssrn.com/sol3/cf_dev/AbsByAuth.cfm?per_id=5559333)^a^, [Dengjie Luo](https://papers.ssrn.com/sol3/cf_dev/AbsByAuth.cfm?per_id=5559331)^a^, Pingwu Liu ^b^, Yun Li^a^*, Peng Chen ^a^*

^a^ Guangxi Key Laboratory of Agro-environment and Agric-products Safety, Key Laboratory of Plant Genetics and Breeding, College of Agriculture, Guangxi University, Nanning 530004, PR China

^b^ Sanya Nanfan Research Institute of Hainan University, Sanya 572025, China

**Table S1:** List of primer used in this study. These primers were designed from Cd transporter genes, antioxidant enzyme gene and Cd-resistance genes.

| **Sr. No.** | **Gene name** | **Code** | **Type** | **Primer sequences (5'-3')** |
| --- | --- | --- | --- | --- |
| 1 | Cadmium/zinc-transporting ATPase HMA1 | HMA1 | F | ATGGGTGAGCGCTGGATTCT |
|  |  |  | R | ACTGATGGAAGAGCGGCCAAT |
| 2 | PREDICTED: metal transporter Nramp5-like | NRAMP6 | F | ATTGTTCTGTGTTATTGGACCG |
|  |  |  | R | AGAAACCTACGGCATAACCATA |
| 3 | Metal-nicotianamine transporter YSL3 -like protein [ | YSL3 | F | TGCCATCGGCTGTGTTGTAG |
|  |  |  | R | AGTGCGGAGAAACCATCGACA |
| 4 | Catalase | CAT | F | TGCGGCGTAATGAGGACAAGTT |
|  |  |  | R | ATGGAAGGGCAGAAGGCCAAT |
| 5 | Peroxidase | POD | F | ATCTCGACGCTCACCGTATCAT |
|  |  |  | R | ACGTGCATCGTCATCGGATTT |
| 6 | Glutathione S-transferase F6 | GST6 | F | CAGATTACTTCACCTTGGCGGA |
|  |  |  | R | ACTCCAAAGCGACAAACCATCA |
| 7 | Metal tolerance protein 1-like isoform X1 | MTP1 | F | GCGAGAACGGTATCGGTACTTT |
|  |  |  | R | AATGGTCTCCGCATCAGAAAAC |
| 8 | Bifunctional L-3-cyanoalanine synthase/cysteine synthase 1 | CS1 | F | CCCCTAACACGCTTTTTGACTT |
|  |  |  | R | GATCGGTGAGGATTAAATCGGC |
| 9 | Fe(^2+^) transport protein 1-like | IRT1 | F | CATTGGGGTATGTTCACCTCTG |
|  |  |  | R | AACTTGTGCCACGGGTTCTCT |
| 10 | PREDICTED: metal transporter Nramp5-like | NRAMP5 | F | GCTCAACCAAGATGGGACCGTA |
|  |  |  | R | TTATGAATCAACCAGCCGACAA |
| 11 | Histone-3 | His3 | F | GTGGAGTCAAGAAGCCTCACAG |
|  |  |  | R | ATGGCTCTGGAAACGCAAA |
